# Inverted colonic diverticulum of the cecum mimicking a submucosal tumor: a case report

**DOI:** 10.1186/s12876-026-04705-9

**Published:** 2026-03-14

**Authors:** Ruizao Zheng, Yu Yao, Huihong Yu

**Affiliations:** 1 Department of Gastroenterology, the Second Affiliated Hospital, Chongqing Medical University, NO.74 Linjiang Road, Yuzhong District, Chongqing, 400010 China; 2 NO.74 Linjiang Road, Yuzhong District, Chongqing, China

**Keywords:** Inverted colonic diverticulum, Cecum, Submucosal lesion, Colonoscopy, Histopathology, Laparoscopic hemicolectomy

## Abstract

**Background:**

Inverted colonic diverticulum (ICD) is a rare endoscopic finding that often mimics a sessile polyp or submucosal tumor, increasing the risk of misdiagnosis and inappropriate intervention. Accurate identification is crucial to avoid potential complications such as perforation. This case is notable for its unusual location in the cecum and its initial misdiagnosis as a gastrointestinal stromal tumor (GIST).

**Case presentation:**

A 66-year-old man with a 10-year history of chronic constipation underwent routine surveillance colonoscopy, which revealed a 30–40 mm sessile lesion in the cecum near the ileocecal valve with a central depression covered by adherent debris/impacted fecal material and peripheral hyperemia; water-jet irrigation was attempted but failed to adequately clear the adherent material. Biopsies obtained from the peripheral mucosa showed benign mucosal hyperplasia, and contrast-enhanced computed tomography (CT) demonstrated focal cecal wall thickening without definite radiologic evidence of malignancy; however, malignancy could not be confidently excluded given the lesion’s size and indeterminate preoperative assessment. After multidisciplinary team (MDT) discussion and shared decision-making with the patient and family, laparoscopic right hemicolectomy was performed. Gross examination showed a submucosal mass with a narrow-necked cavity connected to the bowel lumen. Histopathology confirmed inverted colonic diverticulum, with no evidence of dysplasia or malignancy. The patient recovered well without complications.

**Conclusions:**

This case highlights the diagnostic challenges of inverted colonic diverticulum, particularly when located in the cecum. Recognition of endoscopic features and cautious interpretation of imaging are essential to prevent unnecessary or harmful procedures. Surgical resection remains a definitive option when malignancy cannot be excluded or endoscopic diagnosis is inconclusive.

**Supplementary Information:**

The online version contains supplementary material available at 10.1186/s12876-026-04705-9.

## Background

 Colonic diverticulosis is a common gastrointestinal condition characterized by sac-like protrusions of the colonic wall and occurs predominantly in Western populations, with a prevalence approaching 50% in individuals over 60 years of age [1]. Although most diverticula remain asymptomatic, complications such as diverticulitis, bleeding, and perforation may occur [2, 3]. Inverted colonic diverticulum (ICD) is an uncommon manifestation in which the diverticular wall becomes inverted into the colonic lumen. ICD has been reported in approximately 0.7 to 1.7% of patients with diverticulosis and demonstrates a slight male predominance and a predilection for the sigmoid colon [4, 5]. The underlying mechanism is not fully understood but is thought to involve transient fluctuations in intra-abdominal or intraluminal pressure, such as those occurring during air insufflation in colonoscopy. These pressure changes may contribute to intermittent inward folding of a pre-existing diverticulum [6].

Its nonspecific endoscopic appearance frequently resembles a sessile or pedunculated polyp, creating substantial diagnostic challenges and increasing the risk of perforation during biopsy or polypectomy [4, 7, 8]. Several endoscopic clues have been proposed to differentiate ICD from polyps, including: [1] Aurora rings: concentric pale rings around the lesion, paler than the surrounding mucosa under white-light and narrow-band imaging; [2] Radiating pillow sign: central indentation with radial folds upon gentle pressure using biopsy forceps; [3] Water jet deformation sign: transient flattening or eversion of the lesion during irrigation or air insufflation [5]. Imaging studies such as computed tomography (CT) may reveal a subtle central depression or an umbilicated appearance [9]. Despite these diagnostic clues, preoperative misdiagnosis remains common and may lead to unnecessary or hazardous interventions.

Although advanced endoscopic therapies such as inversion-assisted endoscopic submucosal dissection (ivESD) and endoscopic full-thickness resection (eFTR) have broadened treatment options, segmental colectomy remains the preferred approach for symptomatic or indeterminate cases [5, 10]. Given the limited number of reported cases and the scarcity of evidence-based guidelines, we present a case of cecal ICD associated with chronic constipation and review the relevant literature to enhance understanding of its clinical features, diagnostic strategies, and management approaches.

## Case presentation

**Fig. 1:**
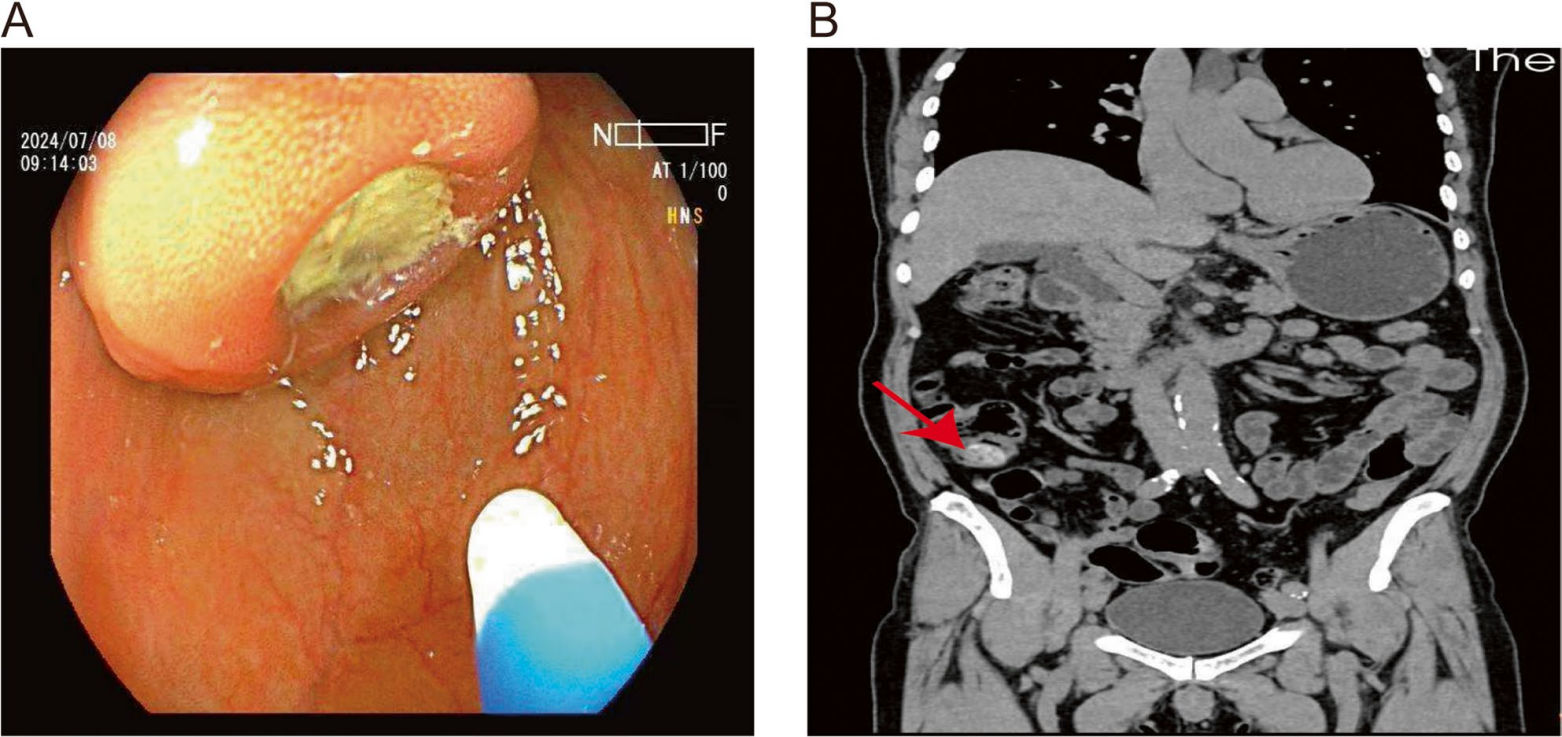
Endoscopic and radiologic features of the inverted colonic diverticulum. **A** Colonoscopic image of a large sessile cecal lesion with a central depression covered by adherent debris/impacted fecal material. **B** Coronal contrast-enhanced CT image showing focal thickening of the cecal wall (arrow) with homogeneous enhancement and no malignant features

**Fig. 2:**
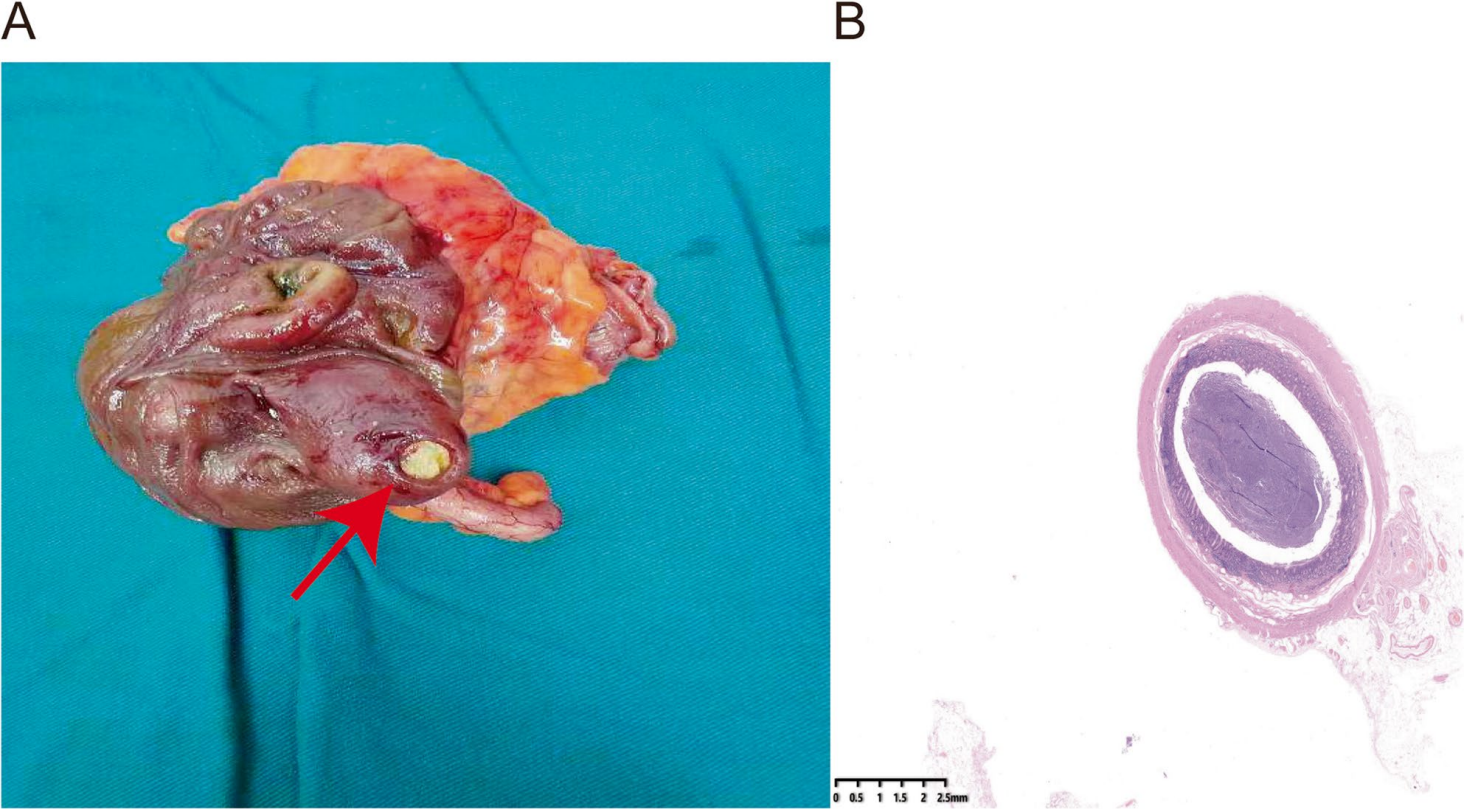
Gross and histologic features of the inverted colonic diverticulum. **A** Resected specimen showing a cecal protruding lesion with a narrow opening and impacted fecal material. **B** Histologic section (hematoxylin and eosin [H&E], ×20) showing inverted colonic mucosa and submucosa forming a pouch-like structure, without epithelial atypia

A 66-year-old man with a 10-year history of chronic constipation underwent routine surveillance colonoscopy. His physical examination and laboratory evaluations were unremarkable. He had previously undergone colonoscopies in 2021 and 2022, during which multiple colorectal adenomatous polyps were detected and removed. During the current examination, colonoscopy revealed a submucosal-appearing sessile lesion measuring approximately 30 to 40 mm in the cecum and proximal ascending colon, near the ileocecal valve, with a central depression covered by adherent debris/impacted fecal material and peripheral mucosal erythema, without active bleeding or an identifiable stalk (Fig. 1A). Water-jet irrigation was attempted to remove the adherent material, but the effect was limited and the material could not be adequately cleared. Biopsies were obtained from the peripheral mucosa rather than the central depressed area and demonstrated polypoid mucosal hyperplasia with focal lymphocytic infiltration, without dysplasia or malignancy. Contrast-enhanced computed tomography (CT) showed localized asymmetric wall thickening of the cecum and proximal ascending colon, measuring approximately 6 mm, with homogeneous enhancement (Fig. 1B). No definite umbilicated appearance was noted. Although the CT scan did not reveal serosal irregularities suggestive of malignancy, the overall preoperative assessment remained indeterminate, and malignancy could not be confidently excluded. Mini-probe endoscopic ultrasound (EUS) was temporarily unavailable due to a technical malfunction of the equipment. The case was discussed at a multidisciplinary team (MDT) meeting, and after shared decision-making with the patient and family, in accordance with the patient’s strong preference for definitive diagnosis and treatment, laparoscopic right hemicolectomy with ileocolic anastomosis was performed. Gross examination of the resected specimen revealed a grayish-brown submucosal lesion in the cecum measuring 2.5 × 2.0 × 1.6 cm. A cystic cavity beneath the mucosal surface communicated with the bowel lumen through a narrow neck (Fig. 2A). Histopathologic evaluation demonstrated that the cavity was lined with non-atypical colonic epithelium, consistent with an inverted colonic diverticulum (Fig. 2B). The postoperative course was uneventful, and the patient recovered well.

### Literature review

A literature review covering the years 2015 to 2025 identified nine publications describing 24 patients with a total of 29 ICD lesions (Table 1). The mean patient age was 60.9 years, and most patients were male (78.2%). The sigmoid colon was the most frequently involved site (58.6%, 17 of 29 lesions), followed by the transverse colon (*n* = 5) [4, 11], ascending colon (*n* = 4) [2, 4, 12, 13], descending colon (*n* = 2) [4, 5], and cecum (*n* = 1) [4]. Only three lesions exhibited a pedunculated morphology on endoscopy. These included one ICD with an adenomatous appearance, one resembling an adenomatous polyp with a hard, hollow head, and one presenting as a polyp-like lesion with a long, thick stalk and an irregular, villous and velvety red surface [5, 11, 14]. The remaining lesions displayed sessile or polypoid morphology, typically with smooth pink mucosa, central depression, or concentric ring patterns. Regardless of their appearance, all ICDs showed a soft, compressible, hollow quality on endoscopic probing [11]. Lesion size appeared to correlate with morphology. Pedunculated ICDs measured 20 × 9 mm and 40 × 20 mm and were noticeably larger than sessile lesions, which had a mean diameter of 10.6 mm (range, 3 to 40 mm). Reported complications of ICD were rare. Only one case of intussusception caused by a large 38 mm ICD was documented by Zhang et al. in 2018 [2]. Diagnosis in most cases relied on characteristic endoscopic findings, including the radiating pillow sign, inversion of the lesion with forceps or air insufflation, the water-jet deformation sign, and the presence of Aurora rings. Only five cases were confirmed pathologically through biopsy [2, 5, 11, 15, 16]. Endoscopic treatment was reported in six cases, including diverticulectomy and ablation, although one patient experienced perforation following polypectomy with hot biopsy forceps [4, 5, 11, 14, 15]. Two patients with ICDs located at the colonic flexures underwent hemicolectomy and had a favorable postoperative recovery [2, 5].

**Table 1:** ICDs epidemiological and endoscopic features

	Cases	Age	Sex	Location	Size	Appearance	Complications	Diagnosis Mode	Treatment
Pinho2015 [14]	1	59	M	sigmoid colon	NA	pedunculated polyp, adenomatos	none	based on appearance	the ligate-and-let-go technique of resectio
Canakis 2017[11]	1	50	M	transvere colon	20 × 9 mm	pedunculated polyp; not soft and empty head with adenomatos appearance	none	tissue biopsy	snare cautery using a Roth net
Mocanu 2018[17]	1	65	M	sigmoid colon	NA	sessile lesion, normal mucosa	none	Aurora rings	none
Zhang 2018 [2]	1	62	W	ascending colon	3.8 cm	none	Intussusception	tissue biopsy	right hemicolectomy
Zimmer 2018 [12]	1	57	NA	right flexure	5 mm	sessile, polypoid, normal mucosa on WLI, BLI, LCI	none	Aurora rings, application of gentle pressure with forceps led to reduction	none
Gulaydin 2021 [4]	19	61.7(47–90)	3 W 11 M	12 sigmoid colon; 4 transverse colon; 1 descending colon; 1 ascending colon; 1 cecum	9.4 (3 − 40) mm	polypoid, normal mucosa; rarely erythema or telangectasia	1 perforation	application of gentle pressure with forceps led to reversion, dimple appearance, air insufflation led to recession into the wall, eversion with aspiration, pillow sign	1 polypectomy with hot biopsy forceps
Lee 2021 [13]	1	69	W	ascending colon	8 mm	polypoid, shiny pink mucosa, normal mucosa	none	The Aurora rings sign seemed ambiguous. The lesion became flat with central dimpling following submucosal injection	none
Cocomazzi 2021 [5]	2	52	M	sigmoid colon	40 × 20 mm	pedunculated polypoid, long thick stalk, irregular and villous head, velvety red	none	the water jet deformation, the soft and easily compressible stalk	the hot snare technique
		75	M	left colon flexure	20 mm	bulging lesion, submucosal lesion, 8–10 mm central depression, villous	none	tissue biopsy	left hemicolectomy
Badawi 2024 [15]	1	56	M	sigmoid colon	NA	shiny mucosal lesion, a polyp with concentric rings around it and no stalk	None	tissue biopsy	mono-polar ablation via tip of snare
Sholi2025 [16]	1	40	M	sigmoid colon	NA	a small polyp	diverticulitis	tissue biopsy	piece-meal polypectomy

## Discussion and conclusions

This case is notable for its uncommon cecal location near the ileocecal valve and proximity to the appendiceal orifice, producing an endoscopic appearance closely resembling a GIST. Because of the lesion’s location, sessile configuration, and uncertain preoperative diagnosis, a right hemicolectomy was performed, and histopathology confirmed ICD. The diverticulum size in this case was consistent with previously reported ICDs, which range from approximately 0.2 cm to 4 cm [18, 19]. The patient’s long-standing constipation likely contributed to chronically elevated intraluminal pressure and abnormal colonic motility, factors known to facilitate inversion of a diverticulum [5]. Diverticula in the ileocecal region are particularly prone to stool impaction and disordered peristalsis, which can promote inward folding and may lead to complications such as intussusception [20].

An additional diagnostic pitfall highlighted by this case is the discrepancy between endoscopic and CT findings. ICD can present as a prominent intraluminal, subepithelial-appearing lesion on colonoscopy, whereas cross-sectional imaging may show only subtle focal wall thickening rather than a discrete mass. Such discordance should prompt consideration of ICD and careful correlation of endoscopic signs (e.g., central depression with adherent material, water-jet deformation) with imaging findings, to avoid unnecessary or hazardous endoscopic interventions. While MRI and abdominal ultrasound are valuable tools in assessing certain gastrointestinal lesions, their ability to differentiate benign from malignant submucosal lesions in the ileocecal region remains limited. These modalities often fail to provide sufficient detail to confidently exclude malignancy, especially in small or indeterminate lesions. Therefore, they were not prioritized in this case, where the lesion’s size and location required more definitive diagnostic methods. Gulaydin et al. reported a case of colonic perforation caused by diverticulum resection using hot biopsy forceps in 2021 [4]. Therefore, recognition of characteristic endoscopic signs is essential. Currently, the water jet deformation sign is considered a safer and more reliable diagnostic method than biopsy forceps and air insufflation [21]. In our case, water-jet irrigation was attempted to clear the adherent material at the lesion center, but it could not be adequately removed, contributing to diagnostic uncertainty. For a sessile, subepithelial-appearing cecal lesion, the endoscopic differential diagnosis includes lipoma, neuroendocrine tumor, lymphoma, adenocarcinoma with submucosal growth, mesenchymal tumors (including gastrointestinal stromal tumor), inflammatory masses, and inverted colonic diverticulum [2]. Mini-probe EUS may help characterize suspected subepithelial lesions and support the diagnosis of ICD; however, it was not available at our center at the time due to a technical malfunction of the equipment. In our patient, given the large size and ileocecal location, benign mucosal biopsy findings and non-specific CT changes were insufficient to establish a single preoperative diagnosis; therefore, the lesion was approached as an indeterminate cecal mass in which malignancy could not be confidently excluded.

Previous reports also support the clinical significance of large right-sided/ileocecal ICD. Hajime et al. reported that malignant colonic tumors may arise in association with ICD [10]. Notably, Zhang et al. described a similarly large ICD in the ileocecal/ascending colon region complicated by intussusception [2], underscoring that even when ICD is suspected, large lesions in this location may warrant proactive management. These findings emphasize the importance of timely management. Although most ICDs in the literature have been treated endoscopically, including reduction using forceps or snare techniques or reduction assisted by submucosal injection, these procedures carry risks such as incomplete reduction, perforation, and recurrence [22]. Segmental colectomy has been reported in a small number of cases. Currently, no consensus exists regarding the choice between endoscopic and surgical management. Endoscopic treatment is generally preferred for small, asymptomatic, and clearly diagnosed ICDs, as well as for early neoplastic changes arising on pedunculated ICDs. Surgical intervention should be considered for large or sessile ICDs, lesions with poor endoscopic visibility, those complicated by obstruction, bleeding, or intussusception, and cases in which malignancy cannot be excluded [5]. Importantly, we do not advocate surgical resection for all ICDs. When ICD is confidently diagnosed and the lesion is small and asymptomatic, conservative management or endoscopic reduction may be appropriate; in our case, the large size, ileocecal location, limited endoscopic visibility due to adherent material, and persistent diagnostic uncertainty justified a more proactive approach.

In older adults with chronic constipation, the presence of an unusual or atypical polypoid lesion should raise suspicion for ICD. A comprehensive endoscopic assessment, complemented by appropriate imaging studies, is essential for establishing an accurate diagnosis. Although surgical management has been reported less frequently in the literature, remains an important definitive option in selected cases. Advances in endoscopic techniques may expand therapeutic options in the future, although long-term data regarding their safety and clinical outcomes remain limited. This report is limited by its single-case nature. In addition, preoperative EUS could not be performed due to temporary equipment malfunction, and mucosal biopsies may not fully reflect submucosal pathology, which contributed to the preoperative diagnostic uncertainty.

## Supplementary Information

Supplementary Material 1.

## Data Availability

Data sharing is not applicable to this article as no datasets were generated or analysed during the current study.

## Abbreviations

ICD: Inverted colonic diverticulum
CT: Computed tomography
GIST: Gastrointestinal stromal tumor
ivESD: Inversion-assisted endoscopic submucosal dissection
eFTR: Endoscopic full-thickness resection
H&E: Hematoxylin and eosin
EUS: Endoscopic ultrasound
WLI: White-light imaging
BLI: Bluelight imaging
LCI: Linked color imaging
NA: Not available
