# Supplementary material for: Inverted colonic diverticulum of the cecum mimicking a submucosal tumor: a case report
Source: BMC Gastroenterol. 2026 Mar 14;26:202. doi: 10.1186/s12876-026-04705-9 (PMC13047800; doi:10.1186/s12876-026-04705-9)
Supplement: Supplementary file 1 — Supplementary Material 1. [file 12876_2026_4705_MOESM1_ESM.pdf]

CARE Checklist of information to include when writing a case report

CARE case report guidelines

BY-NC-ND

Topic	Item	Checklist item description	Reported on Line
Title	1	The diagnosis or intervention of primary focus followed by the words "case report"	Title page
Key Words	2	2 to 5 key words that identify diagnoses or interventions in this case report, including "case report"	Abstract section-key words
Abstract (no references)	3a	Introduction: What is unique about this case and what does it add to the scientific literature?	Background
	3b	Main symptoms and/or important clinical findings	Case Presentation
	3c	The main diagnoses, therapeutic interventions, and outcomes	Case Presentation
	3d	Conclusion—What is the main "take-away" lesson(s) from this case?	Discussion and Conclusions
Introduction	4	One or two paragraphs summarizing why this case is unique (**may include references**)	Discussion and Conclusions
Patient Information	5a	De-identified patient specific information.	Case Presentation
	5b	Primary concerns and symptoms of the patient.	Case Presentation
	5c	Medical, family, and psycho-social history including relevant genetic information	Case Presentation
	5d	Relevant past interventions with outcomes	Literature Review
Clinical Findings	6	Describe significant physical examination (PE) and important clinical findings.	Case Presentation
Timeline	7	Historical and current information from this episode of care organized as a timeline	Not applicable
Diagnostic Assessment	8a	Diagnostic testing (such as PE, laboratory testing, imaging, surveys).	Case Presentation
	8b	Diagnostic challenges (such as access to testing, financial, or cultural)	Case Presentation
	8c	Diagnosis (including other diagnoses considered)	Case Presentation
	8d	Prognosis (such as staging in oncology) where applicable	Case Presentation
Therapeutic Intervention	9a	Types of therapeutic intervention (such as pharmacologic, surgical, preventive, self-care)	Case Presentation
	9b	Administration of therapeutic intervention (such as dosage, strength, duration)	Not applicable
	9c	Changes in therapeutic intervention (with rationale)	Case Presentation
Follow-up and Outcomes	10a	Clinician and patient-assessed outcomes (if available)	Case Presentation
	10b	Important follow-up diagnostic and other test results	Not applicable
	10c	Intervention adherence and tolerability (How was this assessed?)	Not applicable
	10d	Adverse and unanticipated events	Not applicable
Discussion	11a	A scientific discussion of the strengths AND limitations associated with this case report	Discussion and Conclusions
	11b	Discussion of the relevant medical literature **with references**.	Literature Review
	11c	The scientific rationale for any conclusions (including assessment of possible causes)	Discussion and Conclusions
	11d	The primary "take-away" lessons of this case report (without references) in a one paragraph conclusion	Discussion and Conclusions
Patient Perspective	12	The patient should share their perspective in one to two paragraphs on the treatment(s) they received	Not applicable
Informed Consent	13	Did the patient give informed consent? Please provide if requested	Yes ✓ No ☐
